# Supplementary material for: Amorphous Inclusion Complexes: Molecular Interactions of Hesperidin and Hesperetin with HP-Β-CD and Their Biological Effects
Source: Int J Mol Sci. 2022 Apr 4;23(7):4000. doi: 10.3390/ijms23074000 (PMC9000012; doi:10.3390/ijms23074000)
Supplement: Supplementary file 1 [file ijms-23-04000-s001.zip › ijms-1638565-supplementary.pdf]

International Journal of *Molecular Sciences*

MDPI

Article

Amorphous Inclusion Complexes: Molecular Interactions of Hesperidin and Hesperetin with HP-β-CD and Their Biological Effects—Supplementary Material

Kamil Wdowiak [1],Natalia Rosiak [1], Ewa Tykarska [2], Marcin Ożarowski [3], Anita Płazińska [4], Wojciech Płaziński [5], and Judyta Cielecka-Piontek [1,*]

[1] Department of Pharmacognosy, Poznan University of Medical Sciences, Rokietnicka 3, 60-806 Poznan, Poland; kamil.wdowiak@student.ump.edu.pl; nrosiak@ump.edu.pl; jpiontek@ump.edu.pl
[2] Department of Chemical Technology of Drugs, Poznan University of Medical Sciences, Grunwaldzka 6, 60-780 Poznan, Poland; etykarsk@ump.edu.pl
[3] Department of Developmental Neurology, Poznan University of Medical Sciences, Przybyszewski 49, 60-355 Poznan, Poland; zarowski@ump.edu.pl
[4] Department of Biopharmacy, Faculty of Pharmacy, Medical University of Lublin, Chodzki 4a, 20-093 Lublin, Poland; anita.plazinska@umlub.pl
[5] Jerzy Haber Institute of Catalysis and Surface Chemistry, Polish Academy of Sciences, Niezapominajek 8, 30-239 Krakow, Poland; wojtek_plazinski@o2.pl
* Correspondence: jpiontek@ump.edu.pl

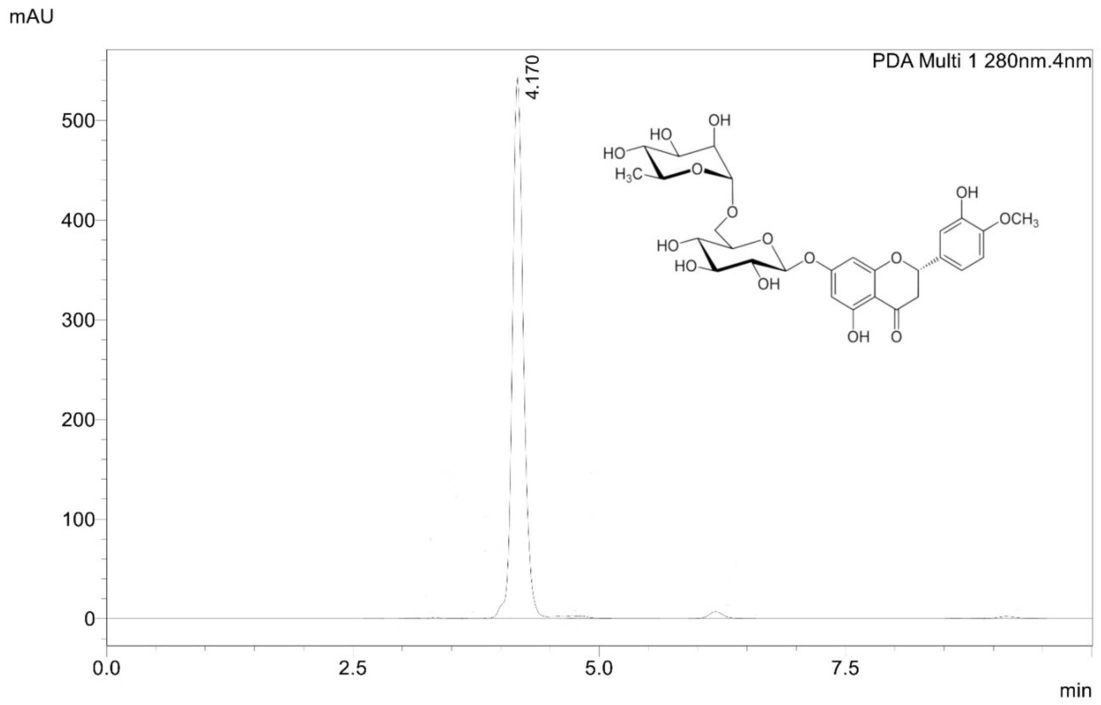

(a)

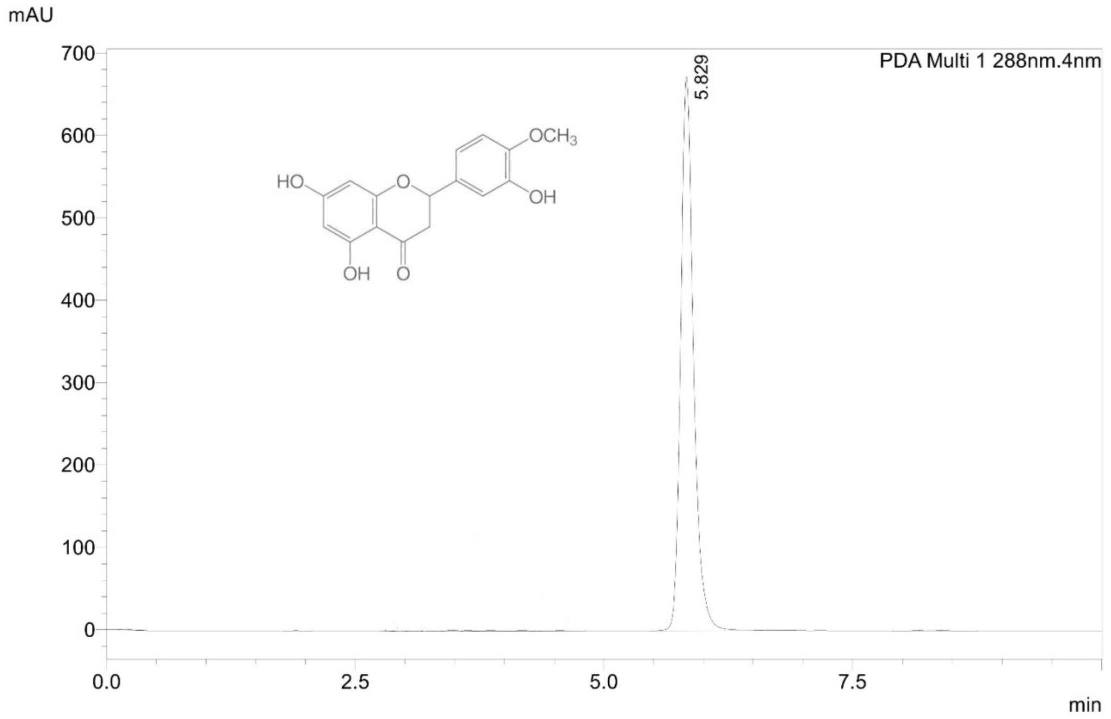

(b)

Figure S1. Chromatograms of Hed (a) and Het (b) for the developed method.

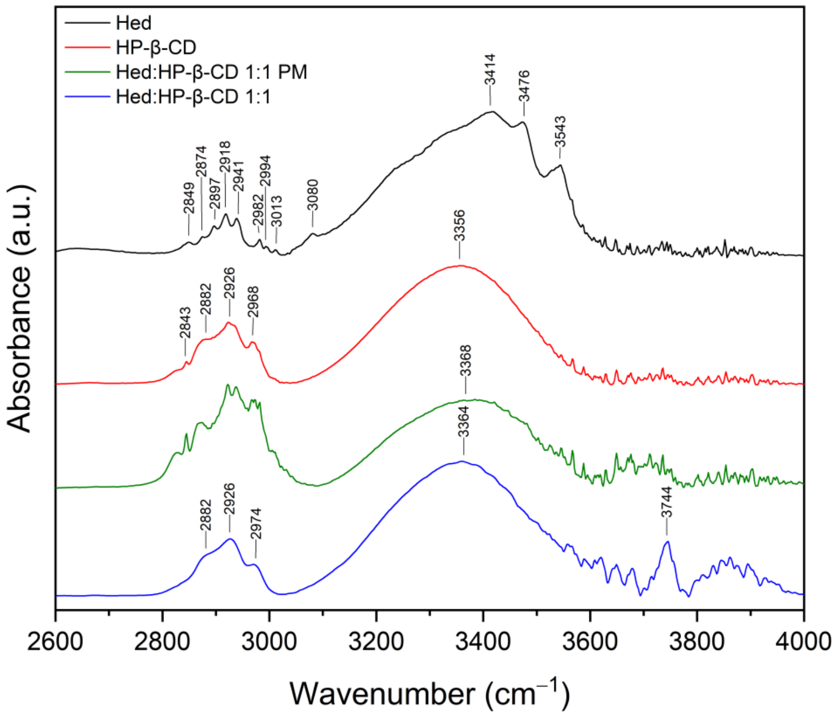

(a)

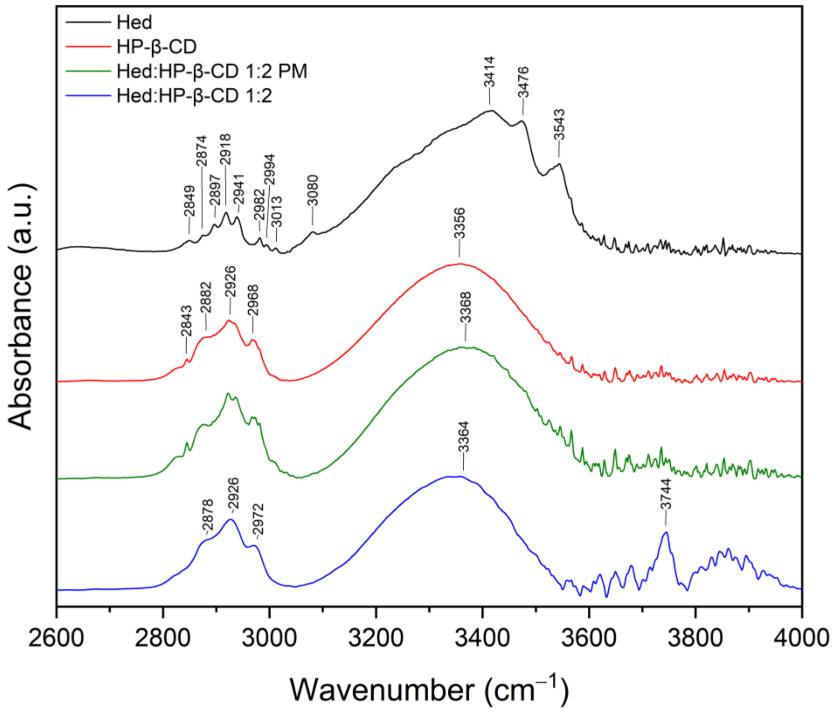

(b)

Figure S2. The results of FT-IR analysis of Hed (black), HP-β-CD (red), Hed:HP-β-CD 1:1 physical mixture (green), Hed:HP-β-CD 1:1 physical mixture systems (blue) (a) and Hed (black), HP-β-CD (red), Hed:HP-β-CD 1:2 physical mixture (green), Hed:HP-β-CD 1:2 physical mixture systems (blue) (b) range 2600–4000 cm^{-1}.

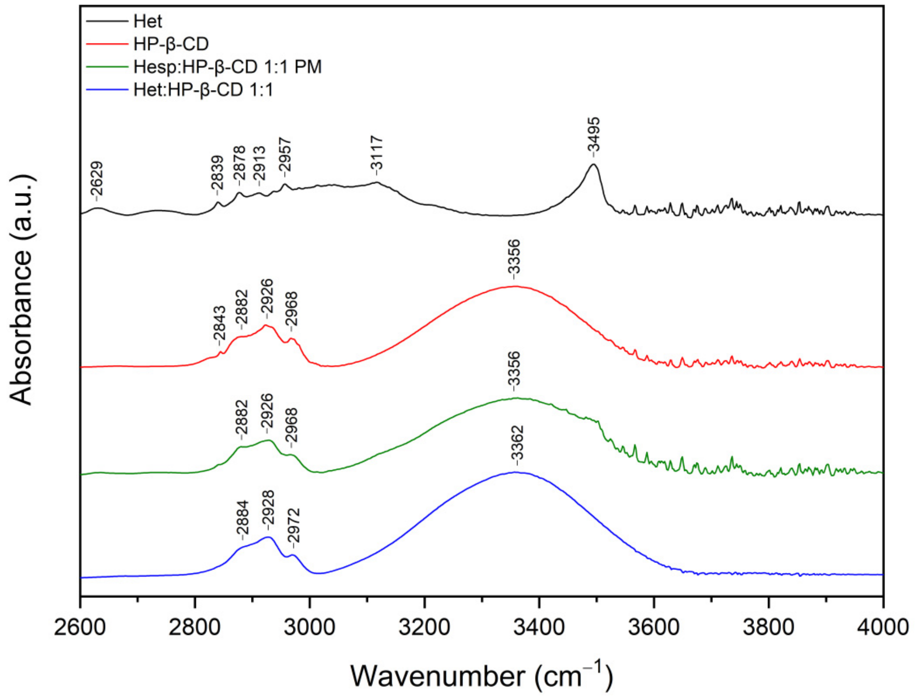

(a)

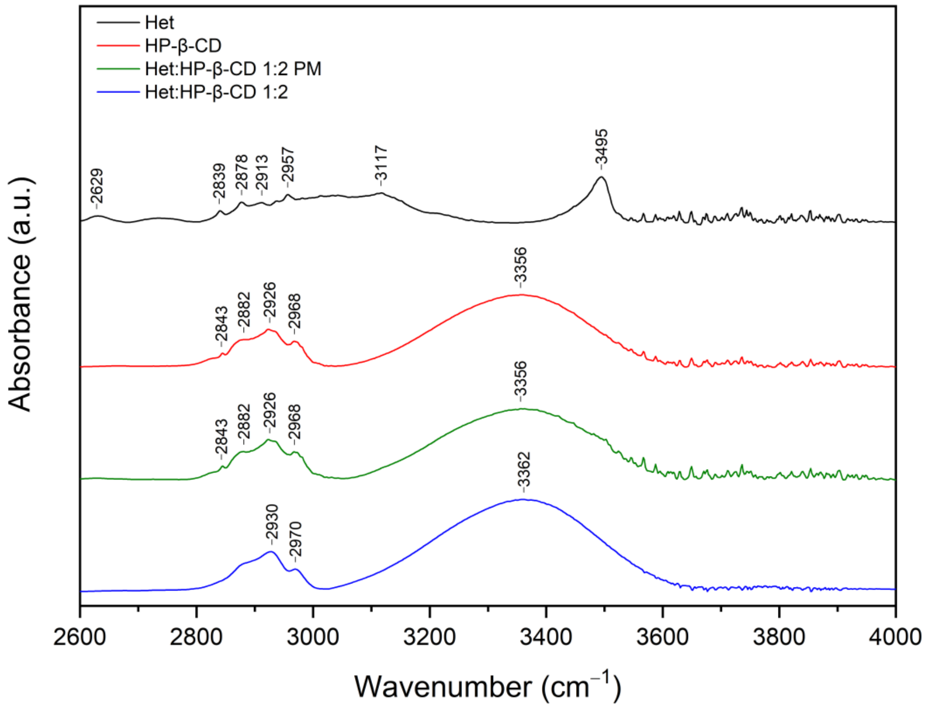

(b)

Figure S3. The results of FT-IR analysis of Het (black), HP-β-CD (red), Het:HP-β-CD 1:1 physical mixture (green), Het:HP-β-CD 1:1 physical mixture systems (blue) (a) and Het (black), HP-β-CD (red), Het:HP-β-CD 1:2 physical mixture (green), Het:HP-β-CD 1:2 physical mixture systems (blue) (b) range 2600–4000 cm^{-1}.

Table S1. Selected experimental peaks (in cm⁻¹) and band assignment of hesperidin, HP-β-CD, Hed:HP-β-CD 1:1 physical mixture, Hed:HP-β-CD 1:1 system, Hed:HP-β-CD 1:2 physical mixture, Hed:HP-β-CD 1:2 system. Legend: def.-deformation, r-rocking, s-stretching, sc-scissoring, t-twisting, w-wagging

**Hed**	**HP-β-CD**	**Hed:HP-β-CD 1:1 PM**	**Hed:HP-β-CD 1:1**	**Hed:HP-β-CD 1:2 PM**	**Hed:HP-β-CD 1:2**	**Band assignment**
419		419	424	419	424	O–H w at rhamnose ring
768		766	-	766	-	breathing B ring
816		816	806	816	-	C–H w at A ring
	847	849	843	847	845	hydrogen bond formation beetwen primary and secondary OH group [1] and the presence of glucopyranose units of 2-hydroxypropyl-β-cyclodextrin in C1 chair conformation [2]
849		849	-	847	-	C–H w at A and B rings
910		-	-	-	-	C–O–C s in glucose ring + C–H r at CH_2 in rutinose
	947	947	947	947	943	the presence of glucopyranose units of 2-hydroxypropyl-β-cyclodextrin in C1 chair conformation [2]
	1018	1015	1016	1016	1013	*
	1034	1034	-	1034	-	C–O s [3]
1034		1034	-	1034	-	C–C s in rutinose ring + C–C s between glucose and rhamnose rings
1049		1051	-	1049	-	O–H r + C–O s at glucose ring
1065		-	-	-	-	C–O s between glucose and rhamnose rings + C–C s in rutinose + O–H r in rutinose
	1080	-	1078	1080	1078	*
1094		1092	-	-	-	C–H t in methyl group + C–O r and C–O s at rhamnose ring
1130		1126	-	1128	-	O–H r and C–H w in methyl group at rhamnose ring
	1150	1152	1150	1153	1144	*
1155		1152	1150	1153	1144	C–O s in rutinose + O–H r and C–H r in rutinose
1182		1180	-	1182	-	O–H r and C–H r at B ring + C–H r and C–H t at C ring
1206		1206	1202	1206	1202	C–O assymetric s in C–O–C between A ring and glucose ring + O–H r at glucose ring + C–H r in all molecule
1242		1238	-	1240	-	C–O–C assymetric s in ring C + C–H r and O–H r at A ring
1277		1275	1269	1277	-	C–O–C s + O–H r at B ring + C–H r at C ring
1298		1300	-	1298	-	C–O s in C–O–C between A ring and glucose ring + O–H r at A ring + C–H r at C ring and glucose ring

*** in the range from 1000–1200 cm⁻¹ with maxima at 1018, 1080 and 1150 cm⁻¹ is defined as coupled vibration of C–O, C–O–C, C–C–O and C–C–C asymmetric valence vibrations [2];**

Table S1. **continued** Selected experimental peaks (in cm^{-1}) and band assignment of hesperidin, HP-β-CD, Hed:HP-β-CD 1:1 physical mixture, Hed:HP-β-CD 1:1 system, Hed:HP-β-CD 1:2 physical mixture, Hed:HP-β-CD 1:2 system. Legend: def.-deformation, r-rocking, s-stretching, sc-scissoring, t-twisting, w-wagging

Hed	HP-β-CD	Hed:HP-β-CD 1:1 PM	Hed:HP-β-CD 1:1	Hed:HP-β-CD 1:2 PM	Hed:HP-β-CD 1:2	Band assignment
	1335	-	1335	1335	1335	C–H vibration [1]
1341		1339	-	1339	-	C–C–C s in A ring + C–H r in all molecule
1356		-	-	-	-	C–H w at C ring + C–H r in all molecule
	1368	1362	1368	1364	1366	C–H vibration [1]
1441		-	-	-	-	C–H w in methoxy group at B ring
	1458	1456	1450	1458	1454	asymmetric C–H deformation vibrations in the plane [2]
1506		1508	1514	1508	-	C–H sc in methyl group at B ring
1518		1518	1514	1518	1512	C–C–C s in A ring + O–H r at A ring + C–H r at C ring
1605		1605	-	1605	-	O–H r at ring A + C=C s in ring A + C=O s at ring C
1645		1647	1639	1647	1639	O–H r at A ring + C=C s in A ring + C=O s at C ring
-	-	-	1742	-	1742	new unidentified band
	2843	2845	-	2845	-	vibration of the –CH and $–CH_2–$ groups [4]
2849		2845	-	2845	-	C–H s in glucose ring
2874		2870	-	2875	-	
	2882	2870	2882	2875	2878	vibration of the –CH and $–CH_2–$ groups [4]
2897		-	-	-	-	C–H at rhamnose ring
2918		2922	-	2923	-	C–H s in methoxy group at B ring
	2926	-	2926	-	2926	C–H stretching of sp3 carbons [5]
2941		2937	-	2937	-	C–H s at rhamnose ring
	2968	2968	2974	2968	2972	CH_3 and CH vibrations [3]
2982		2980	-	-	-	C–H s at rhamnose ring
2994		-	-	-	-	C–H s in methoxy group at B ring
3013		3007	-	3009	-	C–H s in methyl group at rhamnose ring
3080		-	-	-	-	C–H s in methoxy group at B ring
	3356	3368	3364	3368	3364	O–H s [6]
3414		3368	3364	3368	3364	O–H s at A ring
3476		-	-	-	-	O–H s at rhamnose ring
3543		-	-	-	-	O–H s at rhamnose ring
-	-	-	3744	-	3744	new unidentified band

Table S2. Selected experimental peaks (in cm⁻¹) and band assignment of hesperetin, HP-β-CD, Het:HP-β-CD 1:1 physical mixture, Het:HP-β-CD 1:1 system, Het:HP-β-CD 1:2 physical mixture, Het:HP-β-CD 1:2 system. Legend: def.-deformation, r-rocking, s-stretching, sc-scissoring, t-twisting, w-wagging

Het	HP-β-CD	Het:HP-β-CD 1:1 PM	Het:HP-β-CD 1:1	Het:HP-β-CD 1:2 PM	Het:HP-β-CD 1:2	Band assignment
650		650	-	650	-	def. all molecule
739		739	-	-	-	C–H w at A ring
812		814	-	814	-	C–H w at A and B ring
	847	847	851	854	851	hydrogen bond formation beetwen primary and secondary OH group [1] and the presence of glucopyranose units of HP-β-CD in C1 chair conformation [2]
878		878	-	-	-	C–H w at B ring
957		951	-	-	-	breathing A ring + def. C ring + C–C–C s in B ring + C–H r at B ring
	947	951	947	949	947	the presence of glucopyranose units of 2-hydroxypropyl-β-cyclodextrin in C1 chair conformation [2]
	1018	1024	1016	1024	1016	*
1024		1024	-	-	-	def. B ring + –O–C– s in methoxy group at B ring + C–C–C s in A ring
	1034	-	-	1034	-	C–O s [3]
1063		1061	-	-	-	C–C s in C ring
	1080	1082	1080	1080	1080	*
1092		-	-	-	-	C–O–C assymetric s in C ring + C–C–C r in A ring + –O–H r at A ring
1124		1126	1126	1124	-	C–C–C sc in B ring + C–H sc and O–H r at B ring
	1150	1153	1152	1153	1153	*
1169		-	-	-	-	breathing B ring + C–H w and C–H t in methylene group at C ring + –O–H r at A ring
1202		1204	1202	1204	-	–O–H r at A and B ring + C–H r at B ring + C–H w at C ring
1240		1240	-	1240	-	C–H w at ring C + C–H r and –O–H r at A and B ring
1261		1261	-	1261	-	C–H w at C ring + breathing A ring + C–H r and –O–H sc at A ring
1281		1283	-	1282	1277	C–H w at C ring + C–H r + –O–H sc + –C–O s at B ring
1304		1304	-	1306	-	C–H w + C–H w in methylene group at C ring + –O–H sc at B ring
	1335	1339	1333	1339	1337	C–H vibration [1]

*** in the range from 1000–1200 cm⁻¹ with maxima at 1018, 1080 and 1150 cm⁻¹ is defined as coupled vibration of C–O, C–O–C, C–C–O and C–C–C asymmetric valence vibrations [2];**

Table S2. **continued** Selected experimental peaks (in cm⁻¹) and band assignment of hesperetin, HP-β-CD, Het:HP-β-CD 1:1 physical mixture, Het:HP-β-CD 1:1 system, Het:HP-β-CD 1:2 physical mixture, Het:HP-β-CD 1:2 system. Legend: def.-deformation, r-rocking, s-stretching, sc-scissoring, t-twisting, w-wagging

Het	HP-β-CD	Het:HP-β-CD 1:1 PM	Het:HP-β-CD 1:1	Het:HP-β-CD 1:2 PM	Het:HP-β-CD 1:2	Band assignment
1337		1339	1333	1339	1337	breathing B ring + C–C–C s in A ring + C–H r in all molecule
1360		1362	1369	1362	1369	C–H r + C–H w in methylene group at C ring + C–C–C s in B ring + –O–H sc at B ring
	1368	1362	1369	1362	1369	C–H vibration [1]
1400		1400	-	1400	-	breathing B ring + C–C–C asymmetric s in A ring + C–H w at C ring + C–H w in methylene group at C ring
1441		1441	-	1441	-	C–C–C s in B ring + C–H r + C–O–H sc + C–H w in methyl group and C–H r at B ring
	1458	1458	1454	1456	1460	asymmetric C–H deformation vibrations in the plane [2]
1474		1474	-	1474	-	C–O–H sc + C–H r at A ring + C–C s in A ring + C–H r at C ring
1503		1508	1516	1508	-	C–C–C s in B ring + C–O–H sc, C–H w in methyl group and C–H r at B ring
1576		1580	1580	1576	-	C–C–C sc in A ring + C–O–H sc at A ring
1634		1636	1639	1636	1639	C=O s at C ring + C–C–C asymmetric s in A ring
	2843	-	-	2843	-	vibration of the –CH and $–CH_2–$ groups [4]
2839		2843		-	-	C–H symmetric s in methoxy group at B ring
2878		-	-	-	-	C–H symmetric s in methylene group at C ring
	2882	2882	2884	2882	2882	vibration of the –CH and $–CH_2–$ groups [4]
2913		-	-	-	-	C–H s in methoxy group at B ring
	2926	2926	2928	2926	2930	C–H s of sp3 carbons [5]
2957		-	-	-	-	C–H s at B ring
	2968	2968	2972	2968	2970	CH_3 and CH vibrations [3]
	3356	3356	3362	3356	3362	O–H s [6]
3495		-	-	-	-	O–H s at A and B ring

Table S3. Powder diffraction data

Hed			Het		
**Angle**	**d Value**	**Rel. Intensity**	**Angle**	**d Value**	**Rel. Intensity**
7,070°	12,49385 Å	4,2%	7,246°	12,19070 Å	38,0%
7,722°	11,43898 Å	4,1%	10,781°	8,19974 Å	4,5%
8,506°	10,38649 Å	13,1%	13,027°	6,79066 Å	5,1%
11,356°	7,78588 Å	5,6%	14,067°	6,29087 Å	19,5%
12,160°	7,27247 Å	44,1%	14,525°	6,09324 Å	87,5%
13,227°	6,68821 Å	6,3%	15,492°	5,71523 Å	12,3%
13,654°	6,47987 Å	21,3%	15,997°	5,53577 Å	2,3%
15,554°	5,69249 Å	93,2%	16,936°	5,23087 Å	100,0%
16,233°	5,45576 Å	38,1%	17,658°	5,01864 Å	39,2%
17,115°	5,17661 Å	4,2%	18,828°	4,70934 Å	0,9%
18,276°	4,85030 Å	11,3%	20,046°	4,42598 Å	5,6%
18,522°	4,78638 Å	21,4%	20,912°	4,24459 Å	25,5%
19,588°	4,52844 Å	100,0%	21,801°	4,07348 Å	6,0%
20,737°	4,27993 Å	17,6%	22,600°	3,93125 Å	18,4%
21,255°	4,17679 Å	45,7%	22,998°	3,86400 Å	24,0%
22,411°	3,96382 Å	56,9%	23,496°	3,78327 Å	34,0%
23,252°	3,82236 Å	5,1%	24,871°	3,57708 Å	14,5%
23,781°	3,73851 Å	20,0%	25,434°	3,49925 Å	14,2%
24,797°	3,58756 Å	42,3%	26,234°	3,39435 Å	59,4%
25,644°	3,47105 Å	6,7%	27,475°	3,24370 Å	7,2%
26,099°	3,41153 Å	12,3%	28,419°	3,13808 Å	5,9%
26,620°	3,34592 Å	12,6%	29,464°	3,02914 Å	49,6%
27,698°	3,21812 Å	12,3%	31,031°	2,87963 Å	3,6%
28,716°	3,10625 Å	11,9%	31,467°	2,84069 Å	1,4%
29,773°	2,99836 Å	3,5%	31,920°	2,80141 Å	1,5%
30,443°	2,93388 Å	4,3%	32,719°	2,73481 Å	4,9%
31,214°	2,86320 Å	3,9%	33,197°	2,69654 Å	1,0%
32,034°	2,79171 Å	10,8%	34,121°	2,62562 Å	2,8%
32,924°	2,71825 Å	5,6%	35,576°	2,52146 Å	6,0%
34,845°	2,57267 Å	3,1%	36,183°	2,48057 Å	2,5%
36,220°	2,47807 Å	6,3%	36,986°	2,42850 Å	2,1%
37,087°	2,42212 Å	12,3%	37,420°	2,40134 Å	1,8%

Table S3. continued Powder diffraction data

Hed			Het		
**Angle**	**d Value**	**Rel. Intensity**	**Angle**	**d Value**	**Rel. Intensity**
38,184°	2,35506 Å	2,0%	38,148°	2,35718 Å	3,2%
39,671°	2,27011 Å	3,3%	38,630°	2,32890 Å	1,3%
40,295°	2,23641 Å	2,3%	39,865°	2,25954 Å	5,0%
40,697°	2,21521 Å	3,3%	41,463°	2,17606 Å	1,3%
41,614°	2,16851 Å	2,4%	42,658°	2,11780 Å	2,2%
42,658°	2,11780 Å	1,3%	43,780°	2,06613 Å	0,8%
44,137°	2,05022 Å	4,1%	44,726°	2,02459 Å	8,5%

Table S4. Validation parameters of HPLC-DAD methods for concentration determination of Hed and Het

**Hed**	
Parameter	Hed dissolved in 50% DMSO; Injection volume 10 µl
Linearity range (mg·mL^{-1})	0.005 – 1.0
Correlation coefficient (r)	0.9991
a ± S_a	20576576 ± 455212
b ± S_b	insignificant (α=0.05)
LOD (mg·mL^{-1})	0.0379
LOQ (mg·mL^{-1})	0.1147
Retention Time	4.17
**Het**	
Parameter	Het dissolved in 50% DMSO; Injection volume 10 µl
Linearity range (mg·mL^{-1})	0.005 – 1.0
Correlation coefficient (r)	0.9986
a ± S_a	37898529 ± 1041449
b ± S_b	insignificant (α=0.05)
LOD (mg·mL^{-1})	0.0459
LOQ (mg·mL^{-1})	0.1391
Retention Time (min)	5.83

Table S5. The results of fitting factors *f1, f2* analysis

**Compared systems**		***f1***	***f2***
Hed	Hed:HP-β-CD 1:1	1404	15
Hed	Hed:HP-β-CD 1:2	2211	5
Hed	Hed:HP-β-CD 1:1 PM	130	67
Hed	Hed:HP-β-CD 1:2 PM	207	56
Het	Het:HP-β-CD 1:1	621	11
Het	Het:HP-β-CD 1:2	787	6
Het	Het:HP-β-CD 1:1 PM	128	44
Het	Het:HP-β-CD 1:2 PM	199	34
